# Speciation Variation and Bio-Activation of Soil Heavy Metals (Cd and Cr) in Rice-Rape Rotation Lands in Karst Regions

**DOI:** 10.3390/ijerph18031364

**Published:** 2021-02-02

**Authors:** Jiachun Zhang, Guiting Mu, Zhenming Zhang, Xianfei Huang, Hui Fang

**Affiliations:** 1 Guizhou Botanical Garden, Guizhou Academy of Sciences, Guiyang 550004, China; zhangjiachun1988@163.com; 2 Institute of Biology, Guizhou Academy of Sciences, Guiyang 550009, China; mugui6925@163.com; 3 Guizhou Provincial Key Laboratory for Information Systems of Mountainous Areas and Protection of Ecological Environment, Guizhou Normal University, Guiyang 550001, China; hxfswjs@gznu.edu.cn; 4 College of Agriculture, Guizhou University, Guiyang 550025, China; fanghui1988@yeah.net

**Keywords:** rice-rape rotation, soil heavy metals, speciation change, bioactivity, Karst

## Abstract

Heavy metals in soil are in a high background state in Karst areas, and agricultural activities will affect the bioactivity of heavy metals. The heavy metal (Cd and Cr) bioactivity and their activation effects in rice-rape rotation lands in Karst areas were studied based on field experiments and laboratory analysis, and the influencing factors of heavy metal activity were analyzed based on the physical and chemical properties of soil. The results suggest that the residual fraction was the largest and the exchangeable fraction was the smallest for both Cr and Cd in rice-rape rotation lands in Karst areas. During the rice-rape rotation process, Cd and Cr tended to be released from the residual fraction and transformed into the other four fractions. The fractions with high bioactivity, including the exchangeable fraction and carbonate fraction, increased to different degrees. Rice-rape rotation could activate the activity of soil Cd and Cr in Karst areas. It is also revealed that the activity of soil Cd and Cr in Karst areas was closely associated with soil pH and electric potential (Eh). In the 0–20 cm soil layer, Cr showed a significant negative correlation with pH (r = −0.69, *p* < 0.05), while both Cr and Cd showed significant negative correlations with Eh, and the correlation coefficients were −0.85 (*p* < 0.01) and −0.83 (*p* < 0.01), respectively. In the 20–40 cm soil layer, Cr showed significant negative correlations with Eh, and the correlation coefficient was −0.95 (*p* < 0.01). No significant correlation between the activity of soil Cd and Cr and soil mechanical composition was observed. This study revealed that special attention should be paid to changes in pH and Eh in consideration of heavy metal activity in the rice-rape rotation process.

## 1. Introduction

Cultivated land resources are fundamental materials for human survival and development, and they are nonrenewable resources [1]. China’s cultivated land area is 122 million hectares, and the world’s existing cultivated land is 136,911 million hectares. China’s cultivated land area accounts for 7% of the world’s existing cultivated land area. In China, cultivated land resources are limited, and reserve resources are insufficient [2]. The per capita occupancy of cultivated land resources is far below the average level of the world. Since the 1950s, the production performance and utilization value of soil have been declining due to the rapid development of modern industrial and agricultural production, the large application of pesticides and chemical fertilizers, and the continuous invasion of atmospheric dust and sewage on farmland [3]. The degradation of farmland soil quality is a serious threat to food security. Recently, many countries have carried out studies concerning soil quality protection measures and technologies [4]. However, the effect of these efforts is not obvious, and feasible methods are still in the research process [5].

Soil is the foundation of all agricultural activities [6]. Cadmium (Cd) and chromium (Cr) are common heavy metal elements in farmland soils [7]. They may cause various degrees of damage to human tissues and organs via their bioconcentration, bioaccumulation, and biomagnification in food chains [8]. The accumulation of Cd in the human body may lead to teratogenic, carcinogenic, and mutagenic diseases, and the accumulation of Cr may lead to carcinogenic, allergic dermatitis or eczema [9,10]. The total contents of heavy metals in soils can only present the accumulation information of heavy metals in soils, and they cannot be used to predict and evaluate the environmental effects and bioavailability of heavy metals in soils [11]. The proportion of different species of soil heavy metals directly affects the migration, transformation and biological toxicity of heavy metals in soils [12]. In addition, the bioavailability of soil heavy metals mainly depends on their speciation and proportion [13]. The composition of soil is complex, and various factors are associated with the complex and diverse speciation of heavy metals in soils [14]. Soil texture affects the permeability and water content of minerals of different particle sizes and then affects the forms of heavy metals in soil [15]. Soil pH is one of the important physical and chemical properties of soil and plays an important role in the activity of soil microorganisms and the synthesis of organic matter. Soil pH affects the speciation of heavy metals by affecting the soil charge environment, changing the equilibrium point of adsorption-desorption and precipitation-dissolution. Soil organic matter plays an important role in the transformation of different forms of heavy metals in soil [16].

There is currently no uniform definition or analysis method for heavy metal speciation in soils [17]. The most widely used method is the sequential extraction method developed by Tessier et al., in which the heavy metals in soils are divided into five fractions: exchangeable fraction (EXC), carbonate fraction (CAR), iron-manganese oxide bound fraction (OX), organic-bound fraction (OM), and residual fraction (RES). Based on the theory behind this method, the bio-availability order is as follows: EXC > CAR > OX > OM > RES [18]. The toxicity and mobility of heavy metals in soils depend on their speciation to a great extent, among which exchangeable heavy metals have the strongest bioavailability [19]. Some studies have shown that the toxicity of soil heavy metals to plants is different: exchangeable state > reduced state > oxidation state > residual state. Some studies have suggested that exchangeable and reducible fractions can inhibit plant growth, while oxidation and residue are not easily absorbed by plants [20,21]. Some studies also claim that Cr and Cd in the residual fraction of rice rhizosphere soil shows negative correlations with catalase activity. However, soil heavy metal activities are different with different planting patterns, especially under high background conditions [22,23].

Guizhou is a typical region where carbonate rocks are widely distributed in China, and the content of soil heavy metals is significantly higher than that in other regions of China [24]. However, soil heavy metals in Guizhou have low activity, and their bioaccumulation is not high under natural conditions. Along with the development of agricultural activities, the activity of soil heavy metals is gradually stimulated, producing a high-risk area due to heavy metal exposure [25]. At present, the research on heavy metals in cultivated soils is mainly in non-karst areas with a low background. More effort should be made to address the following aspects. First, the accumulation and migration of heavy metals in plants is a dynamic process along with the growth of plants, and studies concerning soil heavy metals should not be limited to maturity periods or other periods. It should be a dynamic process. Second, more studies should be conducted on the activity changes of soil heavy metals with different agricultural activities [26].

Rice-rape rotation is a common planting pattern in China, especially in Guizhou Province. It is of great significance to study the speciation characteristics of soil heavy metals in rotation planting modes [27]. The main aims of this study are as follows: (a) to present a systematic study on the speciation of soil heavy metals in high background areas with low activity; (b) to discuss the effect of the rice-rape rotation planting mode on soil heavy metal speciation and activity; and (c) to study the comprehensive effects of the planting mode (rice-rape rotation) and soil properties on soil heavy metals. We hope to present useful information on heavy metal activity regulation and pollution control with the rice-rape rotation planting mode.

## 2. Materials and Methods

### 2.1. Study Area

The study area (106°44′28″ E, 27°30′11″ N) is located in Shiban Town, Bozhou District, Zunyi City, Guizhou Province. The altitude is approximately 800 m above sea level, the annual average temperature is 14.9 °C, and the annual precipitation is approximately 1020.6 mm. This area has a subtropical monsoon humid climate, and the soil type is calcareous soil and paddy soil. The rape was Youyan-57, and it was a semi-winter recessive genic male sterile hybrid of Brassica napus (national approval number is 2013001). The rice was Yixiang 725, which was bred by crossing Yixiang 1A with Mianhui 725 by the Mianyang Institute of Agricultural Sciences [28]. The general information of the sampled soils is presented in Table 1.

### 2.2. Field Experimental Design

**Figure 1:**
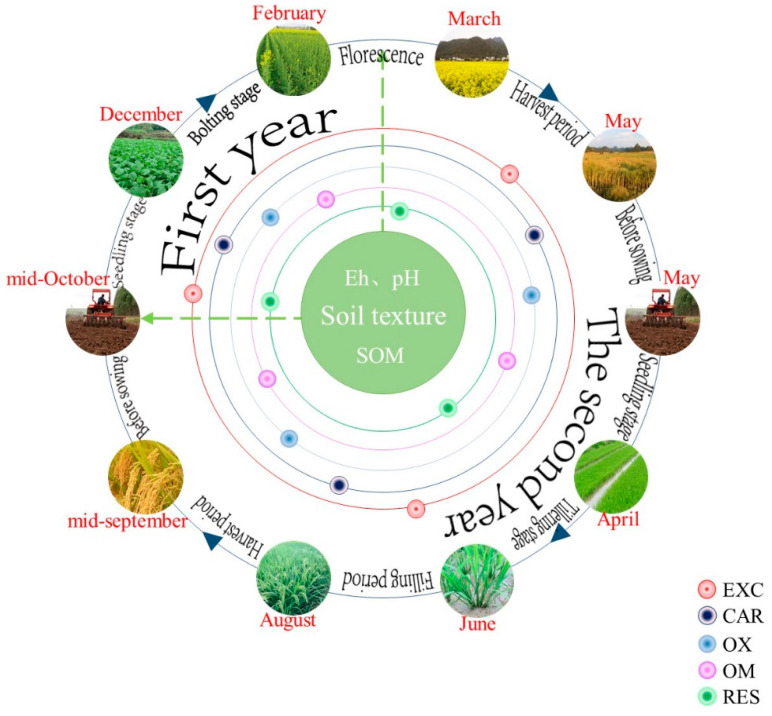
Design of field experiments. (Note: EXC is the exchangeable fraction, CAR is carbonate fraction, OX is iron-manganese oxide bound fraction, OM is organic-bound fraction, RES is residual fraction).

The design of rice-rape rotation was carried out according to local traditional farming methods. Rape was sown in the middle of October. The row spacing was 35 cm, and the seedling spacing was 20 cm. The rape was harvested over ten days late in May of the next year. Rice seedlings were raised in late April, transplanted in early June, and harvested in the middle of September. Sampling activities were carried out before sowing and through four growth periods (regarding rape: seedling stage, bolting stage, florescence stage and harvest period; regarding rice: seedling stage, tillering stage, filling period and harvest period), as shown in Figure 1. Soil depths of 0–20 cm and 20–40 cm were sampled. Five sampling points were set in each sampling unit, and the sampling points were kept away from the ridge and road.

### 2.3. Analytical Procedure

The soil pH was determined by potentiometer method (Soil:Water = 1:2.5). SOC was determined by K_2_Cr_2_O_7_ oxidation at 170–180 °C, and then titration with FeSO_4_. The soil Eh was directly determined by platinum electrode method and soil mechanical composition was determined by simple hydrometer method [9]. The total nitrogen content was determined by Kjeldahl method and total phosphorus content was determined by acid soluble molybdenum antimony anti colorimetric method. The soil bulk density was determined by cylindrical core method [16]. To analyse the total contents of heavy metals, soil samples were digested with HF-HNO_3_-HClO_4_. The heavy metal speciation was separated according to the procedure developed by Tessier et al. [18]. The heavy metal concentration was analyzed using inductively coupled plasma mass spectrometry (ICP-MS 2030 from SHIMADZU Corporation, Japan). To ensure analytical quality, two reference materials were employed: GBW07403 for controlling the total amount analysis and GBW07436 for controlling the speciation analysis. All the recoveries were within the range of 85–105%.

In the present study, soil heavy metal activity was expressed by the heavy metal mobility factor (MF), which was calculated via Equation (1).

(1) $$\mathrm{MF}=\frac{EXC+CAR}{EXC+CAR+OX+OM+RES}\times 100\%$$

## 3. Results and Analysis

### 3.1. Characteristics of Soil Cr and Cd in Rice-Rape Rotation Lands

During the period of rape growth, the contents of Cr in the 20–40 cm soil layer were generally greater than those in the 0–20 cm soil layer except during the harvest period, as listed in Table 2. The contents of Cd in the 20–40 cm soil layer were greater than those in the 0–20 cm soil layer in the seedling and bolting periods, and an inverse phenomenon was observed in the florescence and harvest periods. During the period of rice growth, the contents of Cd in both the 0–20 cm and 20–40 cm soil layers decreased during the growing process. Cr in the 0–20 cm soil layer decreased with the growth process. Cr at 20–40 cm increased during the period from the seedling stage to the tillering stage and then decreased from the tillering stage to the harvest stage.

### 3.2. Variation in Heavy Metal Speciation during the Rice-Rape Rotation Process

**Figure 2:**
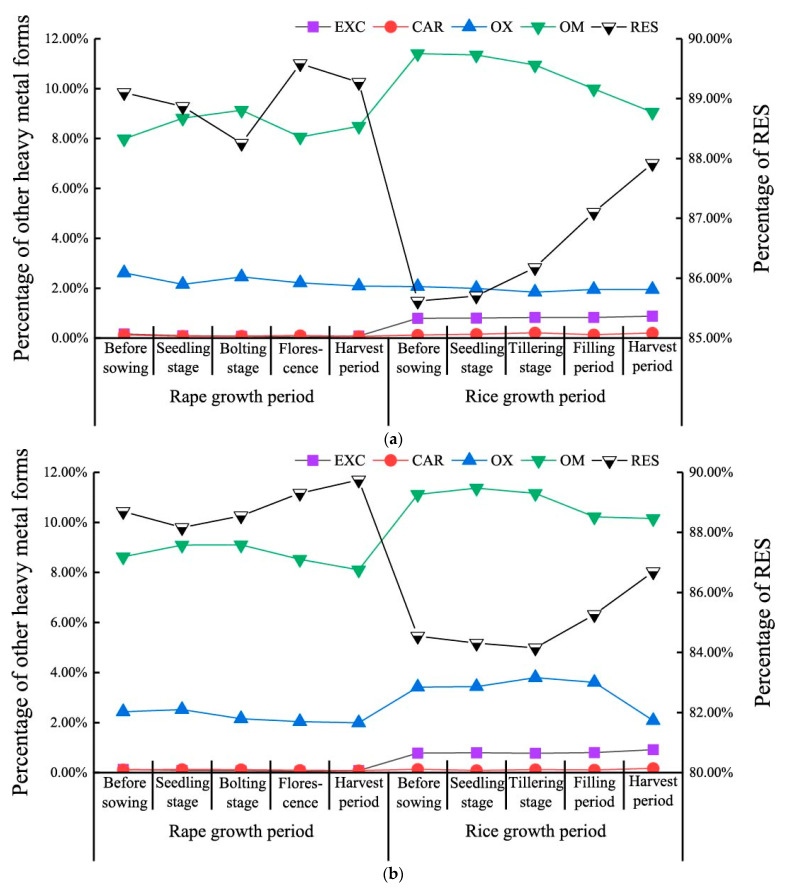
Speciation variation of soil Cr under rape-rice rotation mode. (Notes: (**a**) is 0–20 cm, (**b**) is 20–40 cm, EXC is the exchangeable fraction, CAR is carbonate fraction, OX is iron-manganese oxide bound fraction, OM is organic-bound fraction, RES is residual fraction).

The proportion of soil Cr in the exchangeable and carbonate-bound fractions of the 0–20 cm soil layer during the rice growing period was higher than that during the rape growth period (Figure 2a). Cr in the other three forms changed little under the rice-rape rotation mode (Fe-Mn oxide bounded fraction ranged from 1.84% to 2.61%, organic bounded fraction from 7.99% to 11.40%, and residual fraction from 85.62% to 89.58%). In the 20–40 cm soil layer, the soil Cr in the exchangeable and carbonate-bound fractions was greater in the rice growth period than in the rape growth period (Figure 2b). The soil Cr in the iron and manganese oxide fractions was stable under the rape-rice rotation mode. There was little increase in the rape growing period and a slight decrease in the rise growing period. The variation characteristics of soil Cr in the organic-bound fraction were similar to those in the iron and manganese oxide fractions. However, the variation amplitude of soil Cr in the organic-bound fraction was larger than that in the iron and manganese oxide fractions. The variation characteristic of soil Cr in the residual fraction was opposite to that in the Fe-Mn oxide-bound fraction, and the amplitude of variation was large.

**Figure 3:**
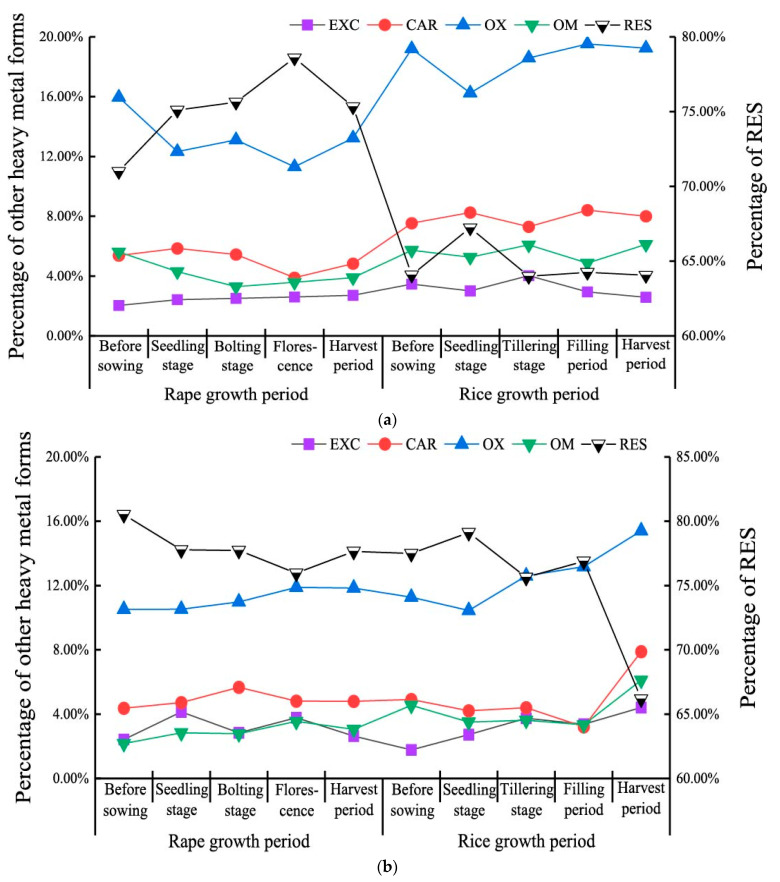
Speciation variation of soil Cd under rape-rice rotation mode. (Notes: (**a**) is 0–20cm, (**b**) is 20–40 cm, EXC is the exchangeable fraction, CAR is carbonate fraction, OX is iron-manganese oxide bound fraction, OM is organic-bound fraction, RES is residual fraction).

During the rice-rape rotation process, most of the soil Cd existed in the residual fraction, accounting for 64.07% to 78.59% (Figure 3a). Following the residual fraction, the soil Cd in the Fe-Mn oxide-bound fraction reached 11.31% to 19.52, and the exchangeable fraction was the lowest fraction, accounting for only 2.03% to 4.03%. The mean value of soil Cd in the exchangeable fraction during the rice growing process (2.46) was smaller than that during the rape growing process (3.21%). The soil Cd in the carbonate fraction increased during the rotation process. Compared with the rape growth process, the proportion of soil Cd in the carbonate fraction during the rice growth process increased by 55.44%. Soil Cd in the Fe-Mn oxide-bound fraction decreased during the rape growing process and increased during the rice growing process. However, the proportion of soil Cd in the Fe-Mn oxide-bound fraction at the end of rotation had little change compared with that before sowing rape. The proportion of soil Cd in the residue fraction during rape growth was higher than that during rice growth. Under the rape-rice rotation mode, the proportion of residual soil Cd in the 20–40 cm soil layer (66.18~80.52%) was the largest, followed by the Fe-Mn oxide-bound fraction (10.44%~15.40%), exchangeable fraction (1.77~4.42%), carbonate fraction (3.21~7.89%), and organic-bound fraction (2.17~6.11%) (Figure 3b). Soil Cd in the exchangeable fraction, carbonate fraction, iron-manganese oxide-bound fraction, and organic-bound fraction presented little variation in the rape-rice rotation process. Soil Cd in these fractions increased. However, soil Cd in the residue fraction decreased significantly during the rice harvest period.

### 3.3. Transport of Heavy Metals

As listed in Table 3, Cr was mainly concentrated in the roots at the seedling and bolting stages of rape, and the transport coefficient in stems and leaves was relatively small (transport coefficient smaller than 1) in these stages. In the florescence period, Cr was transported into stems and flowers, and the transport coefficients were 1.78 and 1.69, respectively. During the harvest period of rape, Cr mainly accumulated in the root parts, and the transport coefficients of Cr in stems, seeds and pods were less than 1. Generally, Cr was transported into different organs in the florescence stage. Rape not only has a strong ability to accumulate Cd but also has a strong ability to transport it. During the period from the seedling stage to the bolting stage, Cd was transported from roots to leaves. At the florescence stage, Cd was transported to leaves and stems, and the transport coefficients were 2.34 and 1.11, respectively. During the harvest period, Cd was transported to stems and pods, and the transport coefficients were 1.43 and 1.53, respectively. In the growing process, Cd was transported from roots to stems and pods.

The transport coefficient of Cr in rice stems decreased at first and then increased during the rice growth process. The transport coefficient of rice leaves to Cr increased gradually from the seedling stage to the filling stage. In contrast, the transport coefficient of rice leaves to Cd decreased gradually from the seedling stage to the filling stage. With the completion of rice filling and the maturation of rice, the contents of the two heavy metals in the rice grain increased. Cr was distributed uniformly in rice and rice husks. Most of Cd was transported into japonica rice. The transport coefficients of all rice organs were smaller than 1. It is suggested that Cd in rice accumulated in the root system, and a small portion was transported into the other organs.

### 3.4. Activation of Soil Heavy Metals in the Rape-Rice Rotation Mode

**Figure 4:**
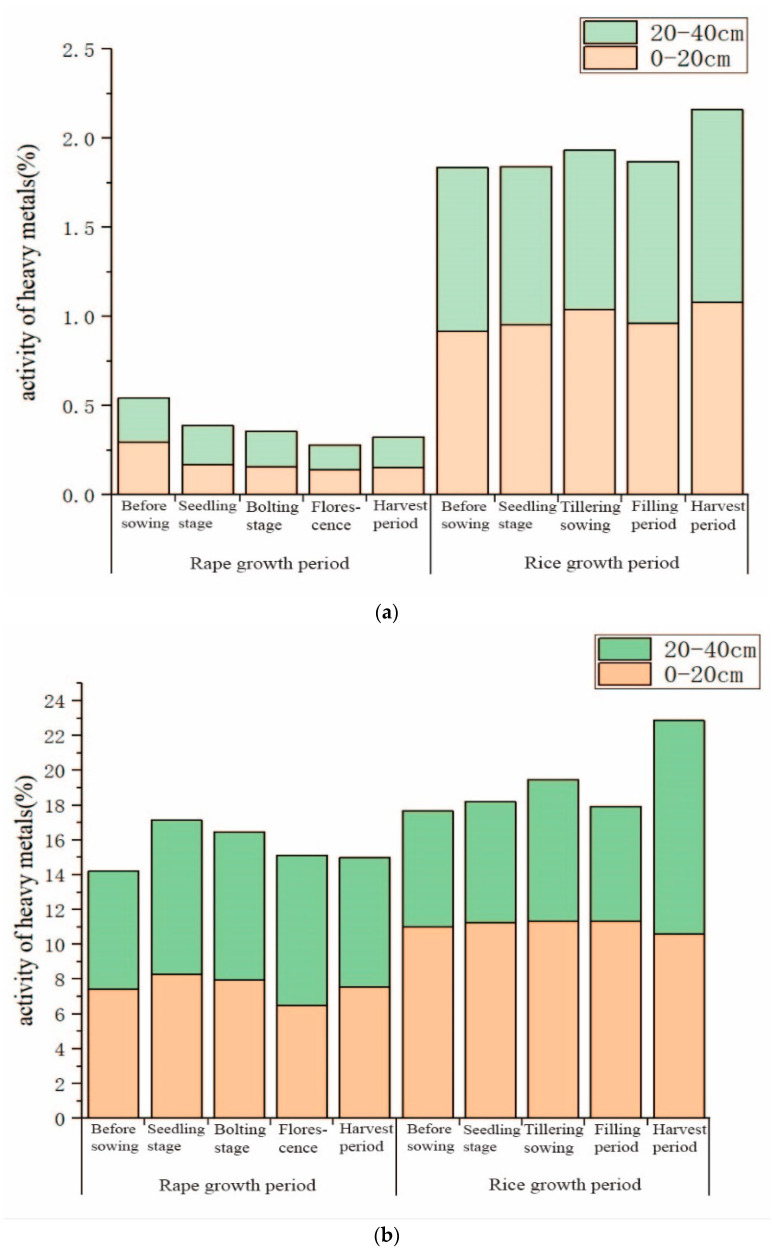
Variation of soil heavy metal activity under the rape-rice rotation (Notes: (**a**) is Cr, (**b**) is Cd).

As shown in Figure 4a, Cr activity in the rice growing process was greatly enhanced compared with that in the rape growing period. The activity of Cr in the 0–20 cm soil layer and 20–40 cm soil layer showed the same variation characteristics. The rice-rape rotation could activate soil Cr. There were some discrepancies between Cd in the 0–20 cm and 20–40 cm soil layers in the rape-rice rotation mode (Figure 4b). In the 0–20 cm soil layer, the activity of Cd increased slightly in the rice growing period. However, it was stable in different growth stages of rice. In the 20–40 cm soil layer, the activity of Cd increased substantially at the end of this rotation (harvest of rice), which changed the previous trend of a small fluctuating decrease.

### 3.5. Effecting Factors of Soil Heavy Metal Activity

In the 0–20 cm soil layer, Cr in the exchangeable fraction was significantly positively correlated with the carbonate-bound fraction (r = 0.81, *p* < 0.01) and organic-bound fraction (r = 0.78, *p* < 0.01) (Table 4), while the exchangeable fraction was negatively correlated with the Fe-Mn oxide-bound fraction (r = −0.73, *p* < 0.01) and residual fraction (r = −0.83, *p* < 0.01). Cr in the organic bound fractions was negatively correlated with the residual fraction (r = −0.98, *p* < 0.01). Generally, Cr in the residual fraction and Fe-Mn oxide-bound fraction in the 0–20 cm soil layer were transformed into the exchangeable fraction, carbonate-bound fraction, and organic-bound fraction. In the 20–40 cm soil layer, Cr in the exchangeable fraction was significantly positively correlated with the Fe-Mn oxide-bound fraction (r = 0.68, *p* < 0.05) and organic-bound fraction (r = 0.86, *p* < 0.01) and negatively correlated with the residual fraction (r = −0.88, *p* < 0.01). Cr in the iron-manganese oxide-bound fraction was significantly correlated with the organic-bound fraction (r = 0.87, *p* < 0.01) and residual fraction (r = −0.92, *p* < 0.01). A negative correlation was also found between Cr in the organic-bound fraction and the residual fraction (r = −0.97, *p* < 0.01). In the rape-rice rotation mode, soil Cr in the residual fraction transformed into the exchangeable fraction, Fe-Mn oxide-bound fraction and organic-bound fraction.

In the 0–20 cm soil layer, no significant correlation between Cd in the exchangeable fraction and in the other four fractions was found (Table 5). Cd in the carbonate-bound fraction was significantly correlated with Cd in the Fe-Mn oxide-bound fraction (r = 0.86, *p* < 0.01), organic-bound fraction (r = 0.67, *p* < 0.05) and residual fraction (r = −0.89, *p* < 0.01). Cd in the Fe-Mn oxide-bound fraction was significantly correlated with the organic-bound fraction (r = 0.85, *p* < 0.05) and residual fraction (r = −0.95, *p* < 0.01). It was also found that Cd in the organic-bound fraction was significantly negatively correlated with the residual fraction (r = −0.87, *p* < 0.01). In the 20–40 cm soil layer, soil Cd in the exchangeable fraction was closely correlated with the residual fraction (r = −0.62, *p* < 0.05), and no significant correlation was found between Cd in the exchangeable fraction and the other fractions. Cd in the carbonate-bound fraction was significantly correlated with Cd in the organic-bound fraction (r = 0.68, *p* < 0.05) and residual fraction (r = −0.81, *p* < 0.01). Cd in the Fe-Mn oxide-bound fraction also correlated with the organic-bound fraction (r = 0.75, *p* < 0.01) and residual fraction (r = −0.92, *p* < 0.01). Cd in the organic-bound fraction was also correlated with the residual fraction (r = −0.88, *p* < 0.01). In the rape-rice rotation mode, the proportion of Cd in the residual fraction was small, and it was probably transformed into the other four fractions.

### 3.6. Correlations between Heavy Metal Activity and Soil Physical-Chemical Properties

In the 0–20 cm soil layer, the activities of Cr and Cd were negatively correlated with fine sand, and the Pearson correlation coefficients were up to −0.89 (*p* < 0.01) and −0.87 (*p* < 0.01), respectively (Table 6). In the 20–40 cm soil layer, the activities of Cr were negatively correlated with fine sand (r = −0.97, *p* < 0.01). There was no close correlation between other size particles and the activity of Cr and Cd in both the 0–20 cm and 20–40 cm soil layers. The activity of Cr was negatively correlated with pH (r = −0.69, *p* < 0.05) and positively correlated with soil organic matter (r = 0.73, *p* < 0.05). Both the activities of Cr and Cd were negatively correlated with Eh, and the Pearson correlation coefficients were −0.85 (*p* < 0.01) and −0.83 (*p* < 0.01), respectively. The activity of Cr was also negatively correlated with Eh in the 20–40 cm soil layer.

## 4. Discussions

### 4.1. Changes in Soil Heavy Metal Speciation in the Rape-Rice Rotation Mode

Some previous studies have shown that the toxicity of heavy metals to plants is different with their speciation [29], and the toxicity of heavy metals is in a different order: exchangeable fraction > carbonate fraction > iron-manganese oxide-bound fraction > organic-bound fraction > residual fraction. Heavy metals in the exchangeable fraction, carbonate fraction and Fe-Mn oxide-bound fraction have high bioactivity and are easily absorbed by plants, inhibiting plant growth [30]. Heavy metals in the organic-bound fraction and residual fraction have low bioactivity and are hardly absorbed by plants, causing little negative effect on plants. A large number of studies have shown that Cr and Cd mainly exist in the residual fraction in natural soil [31]. The proportions of Cr and Cd in the active fraction (nonresidue fraction) are quite different. Some studies have also found that there is a negative correlation between Cd in the residual fraction and catalase activity in rice rhizosphere soils [32]. As two aspects of bioactivity, the bioavailability and migration ability of heavy metals are closely related to their speciation [33]. At present, the bioavailability of heavy metals is usually expressed by the mobility factor, which is the ratio of the sum of heavy metals in the exchangeable and carbonate-bound fractions to its sum in all fractions [34]. The stronger the bioactivity of heavy metals is, the greater the risk of ecological pollution [35]. Based on the present study, the proportion of Cr and Cd in the residual fraction is the largest, and that in the exchangeable fraction is the smallest during the rape-rice rotation process. On the whole, the proportion of Cr and Cd in the residual fraction decrease gradually, and the proportion of these two heavy metals in the other four fractions increase in different states. It is suggested that soil Cr and Cd are transformed into the other four fractions, and the rape-rice rotation mode can activate these heavy metals.

### 4.2. Factors Affecting Soil Heavy Metals during the Rape-Rice Rotation Process

Soil pH is one of the important factors affecting soil activity, and it affects the activity of soil heavy metals in the following three ways [36]. First, the variation in pH changes the number of negative charges on the surface of hydrated oxides, clay minerals and organic matter and then changes the soil adsorption capacity of heavy metals. Second, pH affects the stability of complexes formed with soil organic matter and heavy metals and then affects the bioactivity of soil heavy metals [37]. Third, pH affects the transformation of heavy metals and then changes their activity [38]. Soil mechanical composition (soil texture) refers to the percentage composition of various soil particles of different sizes [39,40,41]. Wilcke et al. reported that there were some significant positive correlations between heavy metal elements and the clay content in soils [42]. The effect of soil organic matter on the activity of soil heavy metals is due to the influence of soil organic matter on the transformation of soil heavy metal speciation [43,44]. In the present study, there is little difference among the clay contents of the soil samples. No significant correlation is observed between heavy metal elements and the clay content [45]. The effect of soil organic matter on the bioactivity of soil heavy metals depends on the properties of soil organic matter, properties of heavy metals, speciation of heavy metals, and other factors, such as soil environmental conditions. Soil Eh is an important index that reflects soil aeration, and the change in soil Eh varies significantly in paddy-upland rotation mode [46,47].

In this study, the results from the correlation analysis between soil heavy metal activity and soil physical-chemical properties suggest that soil heavy metal activity is closely correlated with soil pH, soil Eh, and soil organic matter but not with soil mechanical composition (except fine sand powder). The change in soil pH in the 20–40 cm soil layer is greater than that in the 0–20 cm soil layer in the rotation process. The rape-rice rotation model increases the soil pH value, which may be due to the cultivation of upland crops in the rotated paddy fields. In addition, the long drought return process greatly enhances the soil permeability and effectively prevents secondary soil incubation. The heavy metal ions in low pH circumstances are in a cationic state, and the high concentration of H^+^ has competitive adsorption on heavy metal ions, which affects the exchange adsorption of heavy metal ions. Therefore, this may be the main reason why Cr in the 0–20 cm soil layer is significantly negatively correlated with the soil pH (r = −0.69, *p* < 0.05). Eh is significantly negatively correlated with Cr and Cd activities. In the 0–20 cm soil layer, the activities of the soil heavy metals Cr and Cd are negatively correlated with soil Eh, and the correlation coefficients reach −0.85 (*p* < 0.01) and −0.83 (*p* < 0.01), respectively. In the 20–40 cm soil layer, there is a significant negative correlation between soil heavy metal Cr activity and soil Eh, and the correlation coefficient is up to −0.95 (*p* < 0.01).

## 5. Conclusions

Based on the variation in the activity of soil Cr and Cd, it is found that the proportion of Cr and Cd in the residual fraction decreased gradually, and the proportion of these two heavy metals in the other four fractions (exchangeable fraction, carbonate fraction, iron-manganese oxide-bound fraction and organic-bound fraction) increased in different states. It is suggested that soil Cr and Cd were transformed into the other four fractions, and the rape-rice rotation mode could activate these heavy metals. According to the Pearson correlation analysis, we found that the activity of soil Cr and Cd was mainly associated with soil pH, soil Eh, and soil organic matter, and no significant correlation was found between soil mechanical composition and the activity of soil Cr and Cd, with the exception of fine sand powder and the activity of soil Cr and Cd in the 0–20 cm soil layer and soil Cr in the 20–40 soil layer. In Guizhou, heavy metals are characterized by a high baseline and low bioactivity. However, with the development of agricultural activities, the soil physical and chemical properties have changed, and heavy metal activity has been gradually stimulated. Therefore, Guizhou has become a high-risk area for heavy metal exposure. As mentioned above, rape and rice rotation is the main planting model in China, especially in the Guizhou Province. It is of great importance to develop feasible measures to monitor and maintain soil pH and Eh in rape and rice rotation processes in consideration of heavy metal activity.

## Figures and Tables

**Table 1:** General information of sampled soils.

Soil Layers	Index	SOM (g/kg)	Eh (mV)	pH	Cr (mg/kg)	Cd (mg/kg)	SBD (mg/kg)	TN (g/kg)	TP (mg/kg)
0–20 cm	Mean	56.19	10.20	7.80	56.19	1.41	1.12	1.87	1.58
	Standard deviation	2.38	12.62	0.09	2.38	0.13	0.22	0.08	0.14
	Coefficient of variation (%)	4.24	123.73	1.15	4.24	9.22	19.64	4.28	8.86
20–40 cm	Mean	41.03	29.20	8.07	80.61	1.88	1.18	1.65	1.39
	Standard deviation	2.64	6.72	0.10	1.58	0.06	0.17	0.11	0.12
	Coefficient of variation (%)	6.43	23.01	1.24	1.96	3.19	14.41	6.67	8.63

Note: SOM is soil organic matter; Eh is electric potential; SBD is Soil bulk density; TN is total nitrogen; TP is total phosphorus.

**Table 2:** Characteristics of Cr and Cd in soil during rice rape rotation (mg/kg).

Soil Layers	Heavy Metals	Rape				Rice			
		Seedling	Bolting	Florescence	Harvest	Seedling	Tillering	Filling	Harvest
		Mean ± Standard Division							
0–20 cm	Cr	72.7 ± 2.04	80.6 ± 2.80	74.1 ± 1.04	84.4 ± 1.49	78.3 ± 1.44	77.6 ± 2.73	52.5 ± 3.45	48.1 ± 0.97
	Cd	1.67 ± 0.23	1.73 ± 0.14	1.72 ± 0.09	1.50 ± 0.19	1.81 ± 0.34	1.73 ± 0.09	0.57 ± 0.11	0.48 ± 0.08
20–40 cm	Cr	83.9 ± 1.32	82.0 ± 2.59	77.0 ± 2.75	78.0 ± 2.08	81.3 ± 2.51	89.1 ± 2.97	54.0 ± 2.53	49.2 ± 1.35
	Cd	1.06 ± 0.10	1.25 ± 0.15	1.93 ± 0.14	1.6 ± 0.20	1.59 ± 0.12	1.60 ± 0.12	0.60 ± 0.21	0.53 ± 0.19

**Table 3:** Transport coefficients of heavy metals in their organs under the rape-rice rotation mode.

Crops	Cr					Cd				
	Organs	Seedling	Bolting	Florescence	Harvest	Organs	Seedling	Tillering	Filling	Harvest
Rape (MF)	stem	0.25	0.09	1.78	0.38	stem	0.31	0.22	0.12	0.18
	leaf	0.37	0.10	0.84		leaf	0.17	0.22	0.22	0.29
	flower			1.69		flower			0.09	0.25
	grain				0.42	grain				0.12
	pod				0.44	pod				0.12
Rice (MF)	stem	0.96	0.54	1.11	1.43	stem	0.07	0.06	0.06	0.06
	leaf	1.91	1.23	2.34		leaf	0.23	0.18	0.14	0.12
	flower			0.27		flower			0.09	0.11
	grain				0.49	grain				0.05
	rice husk				1.53	rice husk				0.06

Note: MF is the heavy metal mobility factor.

**Table 4:** Transformation of heavy metal Cr forms in surface soil during rice-rape rotation.

Cr Speciation		EXC	CAR	OX	OM
0–20 cm	CAR	0.81 **			
	OX	−0.73 **	−0.42		
	OM	0.78 **	0.43	−0.59	
	RES	−0.83 **	−0.53	0.57	−0.98 **
20–40 cm	CAR	0.38			
	OX	0.68 *	−0.05		
	OM	0.86 **	−0.23	0.87 **	
	RES	−0.88 **	−0.21	−0.92 **	−0.97 **

Note: EXC is the exchangeable fraction, CAR is carbonate fraction, OX is iron-manganese oxide bound fraction, OM is organic-bound fraction, RES is residual fraction. * significant at 0.05 level, ** significant at 0.01 level.

**Table 5:** Transformation of heavy metal Cd forms in surface soil during rice-rape rotation.

Cd Speciation		EXC	CAR	OX	OM
0–20 cm	CAR	0.41			
	OX	0.53	0.86 **		
	OM	0.42	0.67 *	0.85 **	
	RES	−0.56	−0.89 **	−0.95 **	−0.87 **
20–40 cm	CAR	0.38			
	OX	0.52	0.55		
	OM	0.41	0.68 *	0.75 **	
	RES	−0.62 *	−0.81 **	−0.92 **	−0.88 **

Note: EXC is the exchangeable fraction, CAR is carbonate fraction, OX is iron-manganese oxide bound fraction, OM is organic-bound fraction, RES is residual fraction. * significant at 0.05 level, ** significant at 0.01 level.

**Table 6:** Correlation analysis between soil heavy metal activity and soil physical and chemical properties.

Soil Layer	Index	Mechanical Composition						pH	Eh	SOM
		Clay	FSP	MT	CT	FS	CSM			
0–20 cm	Cr	0.19	−0.89 **	0.50	−0.36	−0.24	−0.57	−0.69 *	−0.85 **	0.73 *
	Cd	0.31	−0.87 **	0.56	−0.38	−0.31	−0.62	−0.63	−0.83 **	0.52
20–40 cm	Cr	0.57	−0.97 **	0.41	−0.53	−0.48	−0.23	0.56	−0.95 **	−0.63
	Cd	−0.31	−0.11	−0.23	0.22	0.35	−0.19	−0.16	−0.02	0.57

Note: FSP is fine sand powder; MT is Medium silt; CT is coarse silt; FS is Fine sand; CSM is Coarse sand and medium sand; SOM is soil organic matter; Eh is electric potential. * significant at 0.05 level, ** significant at 0.01 level.

## Data Availability

No new data were created in this study. Data sharing is not applicable to this article.
